# Editorial: Women in sensory neuroscience 2023

**DOI:** 10.3389/fnhum.2024.1516507

**Published:** 2024-11-20

**Authors:** Carlotta Fossataro, Grace Edwards, F. Nienke Boonstra

**Affiliations:** ^1^Psychology Department, University of Turin, Turin, Italy; ^2^Laboratory of Brain and Cognition, National Institute of Mental Health, Bethesda, MD, United States; ^3^Royal Dutch Visio, National Foundation for the Visually Impaired and Blind, Huizen, Netherlands; ^4^Behavioural Science Institute, Radboud University, Nijmegen, Netherlands; ^5^Department of Ophthalmology, Radboud University Medical Center, Nijmegen, Netherlands

**Keywords:** gender bias against women, sensory neuroscience, sensory processes, sensory deficits, women

Similarly to many fields within science, technology, engineering, and mathematics (STEM), pervasive gender imbalances affect the field of neuroscience. Over the 19^th^ century, the radical belief that women had a weaker intellectual ability than men prevented women from accessing post-graduate courses (Metitieri and Mele, 2022). Nevertheless, some pioneering women overcame these social and cultural barriers and made their mark in the field of neuroscience, producing seminal works. Among them, Maria Mikhailovna Manasseina (1843–1903), one of the first women to graduate in medicine in Europe, for her remarkable contribution to the field of sleep deprivation; Laura Elizabeth Forster (1858–1917) for her comparative studies on degeneration of nerve fibers in birds and mammals; Augustine Marie Cécile Mugnier Vogt (1875–1962), one of the first women admitted to medical school in Paris, for her landmark discoveries in the field of neuroanatomy and neuropathology; Rita Levi Montalcini (1909–2012) for her discoveries of the nerve growth factor, which earned her the Nobel Prize in Medicine in 1986.

Although the situation has gradually improved over the past half-century, with increased awareness of gender bias in science yielding to a slight narrowing of the gender bias, female scientists continue to face challenges and hurdles in their career advancement. Data indicate that more than 50% of neuroscience PhD students are women. However, women comprise < 14% of tenure-track faculty (Sibener et al., 2022). As women progress through their careers, a significant number of women leave the field fostering a sharp increase in the proportion of men in the field and in positions of power. Furthermore, gender imbalance in authorship (Huang et al., 2020), citations (Dworkin et al., 2020), and grant funding (Melnikoff and Valian, 2019) have been reported, thus reinforcing the underrepresentation of women in neuroscience. Further, editorial boards of scientific journals may show a similar gender gap. Indeed, more than 50% of editors are male in 76% of Psychology journals and 88% of neuroscience journals (Palser et al., 2022). Hence, long-standing biases and gender stereotypes continue to hinder women's career advancement and discourage women from pursuing careers in scientific research (Marini and Banaji, 2022). This gender gap hinders not only individual growth but also scientific advancements.

Some initiatives actively support women in neuroscience, such as *Women and Neuroscience* (https://www.sfn.org/initiatives/women-and-neuroscience) founded by the Society for Neuroscience to empower women in the field through resources, networking events, professional opportunities, and awards; *the Women in Neuroscience Repository* (https://www.winrepo.org/) is a project to help in identifying and recommending female neuroscientists for conferences, symposia or collaborations; *Bias Watch Neuro* (https://biaswatchneuro.com/about-bwn/), a website that tracks the women/men ratio of invited speakers at conferences, authors being published in neuroscience journals, and awardees of neuroscience awards; *World Women in Neuroscience* (https://ibro.org/world-women-in-neuroscience/) supported by IBRO is a networking body aimed to promote career development for women neuroscientists across the globe through mentoring and networking activities.

The goal of the “*Women in sensory neuroscience 2023*” Research Topic is to highlight the contributions of female researchers in the field of sensory neuroscience. Through this initiative, we aim to inspire more women to engage in the field of neuroscience and break down the barriers that have historically excluded them from academia and research activities. This Research Topic includes four groundbreaking studies that advance our understanding of sensory processes and contribute to the broader body of knowledge in neuroscience.

The study by Tulimieri and Semrau investigated how aging affects proprioception, the sense of body position and movement. Previous research has shown that aging leads to a decline in proprioceptive accuracy in narrow movement range. Tulimieri and Semrau expand on that by exploring a broad range of movement speeds and distances in younger and older adults. The findings demonstrated that aging significantly impairs proprioceptive accuracy across various movement characteristics, suggesting that proprioceptive errors increase with age and are influenced by the speed and distance of the movement. These insights contribute to a deeper understanding of how aging affects motor control and this could result in interventions to maintain motor function in older adults.

The article by Augière et al. explored whether individuals with fibromyalgia (FM) show altered multisensory integration abilities. Indeed, people with FM often experience alterations in tactile processing, which could disrupt multisensory integration. To this aim they exploited a visuotactile temporal-order judgment task to compare the multisensory integration abilities of 15 individuals with FM and 18 healthy pain-free controls. While the study found no significant differences between the groups, it did reveal that pain intensity in individuals with FM was associated with a reduced weight of tactile information in the multisensory perception. These findings contribute to a growing understanding of how chronic pain affects sensory processing.

Facci et al. presented a rare case study of tactile agnosia, a neuropsychological condition characterized by a selective disruption of the ability to recognize objects through touch, despite intact sensory abilities. The case described involves a 55-year-old woman with corticobasal syndrome (CBS), a neurodegenerative disorder. The patient exhibited difficulty recognizing objects by touch with her right hand, despite normal tactile sensitivity. Over time, her symptoms progressed to include other motor and cognitive impairments, consistent with CBS. This is one of the few documented cases of tactile agnosia in CBS, and the study offers valuable insights into the sensory and cognitive deficits associated with the syndrome. And this information might be used for early diagnostics in CBS.

The study by Hu et al. explored how the brain processes threat-related information, even when we are not consciously aware of it. In 20 participants, in 528 trials, an image (finger or car) was shown to the non-dominant eye with a flickering mask to the dominant eye, to achieve suppression of the target. Trial type, image position, content and sound (with or without car noise) were randomized. The study found that threat-related sounds, such as car engine noises, guided participants' eye movements toward suppressed threat-related images, despite the images being outside of conscious awareness. This research demonstrates the brain's prioritization of threat-related stimuli and highlights the complex interactions between auditory and visual sensory systems in the absence of conscious perception. This study illustrates the possible role of car noise in safe participation in traffic in a future with soundless cars.

Together, these articles show the diverse and impactful research conducted by female scientists in sensory neuroscience. From aging and proprioception to chronic pain and multisensory integration, these studies push the boundaries of our understanding of human sensation and perception. We hope that providing platforms for female researchers, such as a Research Topic dedicated to women, will promote greater gender equality and inspire future generations of women in sensory neuroscience.
